# Prediction at the Discourse Level in Spanish–English Bilinguals: An Eye-Tracking Study

**DOI:** 10.3389/fpsyg.2019.00956

**Published:** 2019-05-03

**Authors:** Carla Contemori, Paola E. Dussias

**Affiliations:** ^1^Department of Languages and Linguistics, University of Texas, El Paso, TX, United States; ^2^Department of Spanish, Italian and Portuguese, Pennsylvania State University, Pennsylvania, PA, United States; ^3^Center for Language Sciences, Pennsylvania State University, Pennsylvania, PA, United States

**Keywords:** bilingual language processing, verb implicit causality, predictive processing, eye-tracking, discourse

## Abstract

In two experiments, we examine English monolinguals’ and Spanish-English bilinguals’ ability to predict an upcoming pronoun referent based on the *Implicit Causality* (IC) bias of the verb. In an eye-tracking experiment, the monolingual data show anticipation of the upcoming referent for NP1-bias verbs. For bilinguals, the same effect is found, showing that bilinguals are not slower than monolinguals at processing the information associated with the IC of the verb. In an off-line experiment, both groups showed knowledge of IC bias information for the verbs used in the eye-tracking experiment. Based on the findings of the two experiments, we show that highly proficient bilinguals have similar online and off-line predictions based on IC verb information than monolingual speakers.

## Introduction

Comprehending sentences in a native language involves the integration of information derived from within the sentence itself and from the linguistic and extra-linguistic context in which the sentence occurs. Mounting evidence has demonstrated that monolinguals use the information that is available to them to set up predictive expectations about what, within a sentence, they will encounter, and where it may be found (e.g., Altmann and Kamide, 1999; DeLong et al., 2005; Pyykkönen and Järvikivi, 2010; Cozijn et al., 2011). Evidence for prediction has been found in a range of psycholinguistic processing domains with monolinguals (e.g., Altmann and Kamide, 1999; Frazier et al., 2000; Kaiser and Trueswell, 2004; Wicha et al., 2004; Van Berkum et al., 2005; Apel et al., 2007; Manabu and Keller, 2017), but recent findings with second language (L2) speakers suggest that during sentence processing, L2 learners have difficulty with the concurrent integration of multiple types of linguistic information, affecting their ability to make predictions about what may come next in a sentence. Recent studies have investigated the ability of L2 speakers to predict upcoming information by using event structure (Grüter et al., 2016), grammatical gender (e.g., Grüter et al., 2012; Dussias et al., 2013; Hopp, 2013) and lexical expectancy (Martin et al., 2013, for a review see Kaan, 2014), with findings showing conflicting results.

Notice that in comparison to previous studies that focused on late L2 learners, the present study tests bilinguals who have been exposed to the two languages in childhood, and for which Spanish is the first acquired (L1) language and English is the second acquired language (L2). The question that we address is whether childhood bilinguals also show weaker prediction as shown in individuals who have late exposure to the L2. Furthermore, prediction during L2 online sentence comprehension at the discourse level – a level at which predictive processing is known to also occur in monolingual speakers – is a domain that has been largely neglected in research (see Kohlstedt and Mani, 2018). The main goal of the experiments presented here, then, is to extend the investigation of predictive processing abilities to a new population of speakers, i.e., bilinguals who have had early exposure to the second language.

In the domain of discourse expectations, we focus on the role of implicit causality (Garvey and Caramazza, 1974) – a feature of interpersonal verbs that denotes causal directionality. To illustrate, in (1) the anaphoric interpretation of the pronoun *she* will vary depending on the verb: verbs such as *frighten* and *confuse* will lead to a subject interpretation (e.g., *Sally* is the preferred referent for *she*), whereas verbs like *love* and *hate* will lead to an object interpretation (e.g., *Mary* is the preferred referent for *she*).

(1) Sally VERBs Mary because *she*..

When the verb’s meaning biases readers and hearers to infer that the cause of the action should be assigned to the subject, the verb is usually referred to as NP1 bias; when the cause is assigned to the verb’s direct object, the verb is referred to as NP2 bias (Ferstl et al., 2011).

A verb’s implicit causality has been demonstrated to have immediate effects during L1 reading (e.g., Caramazza et al., 1977) and L1 listening (e.g., Van Berkum et al., 2007). For example, a visual world eye-tracking study by Cozijn et al. (2011) provides evidence for the activation of implicit causality information even before participants (Dutch native speakers) encounter the causal connective [e.g., *because*, in a sentence like (1)], suggesting that native speakers can make predictions about an upcoming event that is connected with the lexical semantics of the verb in the preceding discourse (see Pyykkönen and Järvikivi, 2010, for similar results in Finnish).

The study of prediction involving pronoun resolution at the discourse level, which is the linguistic context examined here, has the potential to shed light on the kinds of information that bilinguals can or cannot integrate in the L2 to anticipate how a conversation might continue. In the case of Spanish-English bilinguals, implicit causality bias is present in English and Spanish psychological verbs; therefore, cross-linguistic interference is not expected for the processing of implicit causality verbs in the participants’ L2 English (Goikoetxea et al., 2008; Hartshorne et al., 2013). By recruiting L2 speakers who have acquired English in childhood and who are highly proficient in the language and by eliminating cross-linguistic differences, the present study aims to examine how bilinguals use verb bias information to create expectations about upcoming referents in the discourse in the L2.

Below we report the results of two experiments, one using eye-tracking methodology during listening, and one using a sentence completion task, to examine whether L2 speakers activate implicit causality information to make predictions about who will be talked about next in a sentence. Before describing the experiments, we discuss the few offline studies that have investigated the role of implicit causality in L2 processing.

### Prediction in L2 Speakers

Recent research has pointed out that L2 learners may show limits in building up expectations during processing, both at the lexical and the morphosyntactic levels (e.g., Kaan et al., 2010; Lew-Williams and Fernald, 2010; Grüter et al., 2012; Martin et al., 2013). To our knowledge, two recent studies employing offline sentence completion tasks have investigated how discourse information can influence the predictions of L2 learners. Grüter et al. (2016) investigated how Korean and Japanese L2 speakers of English used event structure to predict upcoming referential forms. They elicited story continuations following transfer-of-possession sentences (e.g., John –*Source* handed/was handing a book to Bob-*Goal*. He…), in which either a perfective or imperfective verb was presented (e.g., *handed* vs. *was handing*). The authors found that monolingual speakers of English preferred Source continuations (he = John) in the imperfective than in the perfective condition. Results for the L2 learners showed that referential choice was less consistently influenced by the event structure presented in the prompts. In other words, L2 learners showed reduced ability to create expectations based on discourse information (verb aspect) compared to native English speakers.

Particularly relevant for the purpose of our paper is the study by Cheng and Almor (2017), which looked at the preferences for interpreting pronouns in a group of native speakers of Chinese who were intermediate/advanced learners of English. Participants were presented with two sentence completion tasks in which the implicit causality bias of the verb in the preceding sentence was varied, such that in some cases it favored a subject resolution (i.e., the verb was NP1 bias) and in others it favored an object resolution (i.e., the verb was NP2 bias). This is illustrated in (2) and (3), respectively:

(2) John frightened Henry because he….
(3) John feared Henry because he….

Completions provided by the L2 English learners suggested that they could not use implicit causality information as effectively as native English speakers when selecting the NP referent for the pronoun. In particular, Cheng and Almor (2017) found that L2 learners exhibited a general subject or “first-mention” bias, and could not reverse their expectations even when the connective *because* was changed to *so*, as illustrated in (4). A verb that has an NP2 preference under the implicit causality bias, as the example verb *fear*, is known to elicit an NP1 preference when the connective is changed to *so*. This property of certain psychological experiencer verbs is also known as Implicit Consequentiality.

(4) John feared Henry so he….

One possible explanation for the differences observed between the native speakers and L2 learners is that proficiency and cross-linguistic differences between Chinese and English may have played a role. The authors recruited a group of intermediate L2 learners who were not immersed in the L2 environment (i.e., living in China at the time of testing). Therefore, the amount of exposure to the L2 may have impacted the weaker use of implicit causality information in the participants’ sentence completions. With respect to the cross-linguistic differences, although Chinese has implicit causality bias verbs like English, the number of NP2 bias verbs is smaller in comparison to English (Cheng and Almor, 2017). Since in Chinese there are more NP1 than NP2 bias verbs, Chinese native speakers have less exposure to the type of structure presented in (4) in comparison to English native speakers. Therefore, native Chinese speakers who learn English as an L2 may have a preference for interpreting psychological verbs in English by deploying an NP1 bias, due to their experience with their L1.

In the experiments described here, we employed the eye-tracking methodology (which indexes online processing) and a sentence completion task (which indexes offline processing) to examine discourse-based predictive processes in a group of L1 Spanish speakers who are highly proficient and early exposed to English (their L2). Spanish and English were chosen as the bilinguals’ two languages because the implicit causality bias of Spanish and English verbs are comparable (Goikoetxea et al., 2008; Hartshorne et al., 2013), providing assurance that differences in L1 and L2 verb biases are not responsible for difference in predictive expectations between L2 speakers and L1 of the target language. The participants recruited for our experiments had been exposed to English early in life and were living in an English-speaking environment at the time of testing. Doing this also allowed us to explore the quality of the representations of implicit causality verbs in a population of highly proficient (childhood) bilinguals immersed in the L2. To make online predictions, one pre-requisite is that speakers consistently and efficiently retrieve the verb’s lexical information, including the bias associated with it. If retrieval is less efficient in bilinguals compared to monolinguals, one possible outcome is that it may lead to slower/less consistent online predictions. Given this, one prediction is that even highly proficient L1 speakers of Spanish with early exposure to English may show differences in the time-course of the predictions based on implicit causality verbs. However, another possibility is that highly proficient bilinguals show similar online and offline expectations as monolingual speakers. In this case, the results would suggest that proficiency and exposure to the L2 play an important role in the attainment of prediction abilities.

We first report the results of an eye-tracking study exploring predictions in implicit causality contexts using the visual word paradigm. Then, we present the results of an off-line sentence completion task in which participants provided continuations for sentence fragments in which a declarative sentence introduced two referents and an implicit causality verb, followed by an ambiguous pronoun.

## Experiment 1: Eye Tracking Study

### Participants

Twenty-one L1 English-speaking adults (12 females; mean age 20.5; *SD* = 2) and 23 Spanish–English bilingual speakers (14 females; mean age 21, *SD* = 3.5) were recruited at two large American universities. A Language History Questionnaire (LHQ, Marian et al., 2007) revealed that for the L1 English speaking participants, English was the only language that they spoke proficiently. When knowledge of a second language was reported in the LHQ, the second language had always been learned in school and the participants indicated minimal knowledge of that language. Bilingual participants were highly proficient and had childhood exposure to English, as shown in Table 1. Proficiency in English was measured with a subsection of the *Michigan English Language Institute College English Test* (MELICET), containing 50 multiple-choice questions (30 grammar questions and 20 cloze questions). Only bilingual participants who scored at least 40 out of 50 were included in the group. Participants with a score lower than 40 were discarded (*N* = 8).

**Table 1:** Participant information: Mean (SD).

Self-reported measures		Spanish – L1	English – L2
	Age of exposure (age in years)	0 (0)	6 (4)
	Became fluent (age in years)	5 (2)	11 (5)
	Length of residence in a country where the language is spoken (in years)	13 (8)	14 (8)
	Length of residence in a family where the language is spoken (in years)	21 (4)	10 (10)
	Speaking (1–10)	8 (2)	8 (2.5)
	Listening (1–10)	9 (1)	9 (3)
	Reading (1–10)	8 (2)	8 (2)
	Average daily exposure (%)	56 (14)	44 (14)
Language proficiency MELICET	Score (out of 50)	–	41.3 (2.9)

### Materials

#### Norming of the Implicit Causality Verbs

The IC verbs were chosen based on normed accuracy, measured with a sentence completion task, with sentence fragments composed of a NP in the subject position, an implicit causality verb immediately followed by a second NP, and a *because he* clause, as shown in (9). The pronoun in the sentence was potentially ambiguous as both the preceding referents had same gender:

(9) Kevin apologized to Dave because he…

The sentence completion task was completed by 34 monolingual-English speakers recruited through Amazon Mechanical Turk, and included 34 implicit causality verbs (22 NP1-bias and 22 NP2-bias verbs) chosen from previous psycholinguistic studies (Garnham et al., 1996; Stewart et al., 2000). Participants completed the fragments in a way that sounded natural to them, and indicated which NP referred to the pronoun in their continuation. Twenty-two fillers were included that had similar structure as the experimental sentences, but did not contain an implicit causality verb (e.g., Susan spent the holidays with Mary when she….). The experimental and filler sentences were divided into two lists of 34 sentences each (12 sentences with NP1-bias verbs, 12 sentences with NP2-bias verbs, 22 fillers). The task was programmed as a Qualtrics survey. NP1 and NP2 verbs were selected for the eye-tracking stimuli if at least 70% of the continuations provided were consistent with the verb bias.

#### Structure of the Experimental Stimuli

Materials were designed following Cozijn et al. (2011). Auditory stimuli were 24 sentences consisting of a main *biasing clause* with an implicit causality verb selected from the verb norming and two characters (e.g., “Kevin apologized to Dave”), a subordinate causal *neutral clause* (e.g., “in the evening”) and two subordinate causal *disambiguating clauses* introduced by *because he* (e.g., “because he was scared and because he had insulted him.”). The purpose of the neutral clause was to present a pronoun following a main sentence with an implicit causality verb in an environment with no bias. The main clause contained either an NP1-bias verb (12 sentences) or an NP2-bias verb (12 sentences), and the disambiguating clause was either congruent or incongruent with the implicit causality bias of the verb:

(5) *NP1 Verb-Congruent*: Kevin apologized to Dave in the evening because he was scared and because he had insulted him.
(6) NP1 Verb-Incongruent: Kevin apologized to Dave in the evening because he was scared and because he was insulted.
(7) NP2 Verb-Congruent: Kevin believed Dave yesterday because he was kind and because he showed him the photograph of the crime.
(8) NP2 Verb-Incongruent: Kevin believed Dave yesterday because he was kind and because he had seen a photograph of the crime.

As illustrated in examples (5)–(8), in the experimental stimuli designed for the eye-tracking task, common male names were used for the NP1 and NP2 characters (e.g., Kevin, Dave, Tom). The names were counterbalanced across the stimuli, and appeared in half of the stimuli as NP1 and in the other half as NP2. The NP2 in the main clause was always followed by a distractor clause (i.e., a prepositional phrase or an adverb added to the end of the main clause). Stimuli were counterbalanced for congruency, with half of the NP1-bias stimuli having a congruent disambiguating sentence and the other half having an incongruent disambiguating sentence, leading to the creation of 48 experimental stimuli, divided in four lists, following a Latin Square design.

#### Norming of the Experimental Stimuli

In the experimental sentences, congruency was normed using a Qualtrics survey administered through Amazon Mechanical Turk. For each of the implicit causality verbs, three sentences were constructed. The sentences in each set had the same main clause and a different subordinate causal clause. The subordinate clause always contained the pronoun “he” and was designed to be neutral, congruent, or incongruent with the implicit causality bias of the verb in the main clause. The masculine pronoun was selected following the design of the experiment by Cozijn et al. (2011). To illustrate, the NP1-biasing verb presented in (5) and (6) is repeated in the neutral, congruent and incongruent forms used in the norming task.

(10) Neutral: Kevin apologized to Dave in the evening because he was scared.
(11) Congruent: Kevin apologized to Dave in the evening because he had insulted him.
(12) Incongruent: Kevin apologized to Dave in the evening because he was insulted.

The sentences were divided across six lists, each containing 44 experimental items and 30 filler sentences. Thirty participants took part in the norming study. They were instructed to read the sentences, to answer a two-choice question (“Who does ‘he’ refer to?”), and to make a judgment on their choice (“How sure are you about your choice?”) on a scale from 1 to 7. In the majority of the cases, participants selected the referent consistent with the bias of the verb (congruent condition: NP1 verbs: 92%; NP2 verbs: 96%; neutral condition: NP1 verbs: 62%; NP2 verbs: 52%; incongruent condition: NP1 verbs: 89%; NP2 verbs: 94%). We compared the participants’ answers on the two-choice question task by using mixed-effects logistic regression (Jaeger, 2008) with Congruency (3-levels) as fixed effect. Participants’ choices were coded as 1 if they were congruent with the bias of the verb (NP1 or NP2), and as 0 if they were not congruent with the bias of the verb. The model showed a main effect of Congruency (ß = −2.50, *SE* = 0.52, *t* = −4.812, *p* < 0.0001), indicating more congruent choices in the congruent sentence condition in comparison to the neutral (ß = −3.22, *SE* = 0.50, *t* = −6.049, *p* < 0.0001) or the incongruent sentence condition (ß = 3.15, *SE* = 0.95, *t* = 3.230, *p* < 0.001). Additionally, the main effect revealed more congruent choices in the neutral sentence condition in comparison to the incongruent condition (ß = −0.5, *SE* = 0.14, *t* = −3.498, *p* < 0.0002).

#### Preparation of Auditory Stimuli

Audio stimuli were recorded in a quiet room by a male native English-speaker at a normal pace. To ensure consistency across the audio stimuli, both intensity and pitch were scaled and normalized using a PRAAT script (Boersma, 2001). The stimuli with resynthesized pitch that were determined to be naturalistic by a trained phonologist were selected for further use. To ensure that the pre-disambiguating information was the same in any given congruent/incongruent pair, the disambiguating and post-disambiguating regions from the incongruent stimuli were spliced into their corresponding congruent stimuli using a PRAAT script. Ambient noise (which we will refer to here as a PAUSE for ease of exposition) of 400 ms in length, and which was originally present during the recording of stimuli, was inserted after the distractor and the neutral clause, and at the end of each stimulus, as shown in (13):

(13) Kevin apologized to Dave in the evening PAUSE because he was scared
  PAUSE and because he had insulted him PAUSE

The stimuli were concatenated and care was taken to note any abnormalities in speech in these files.

#### Preparation of Visual Stimuli

**FIGURE 1:**
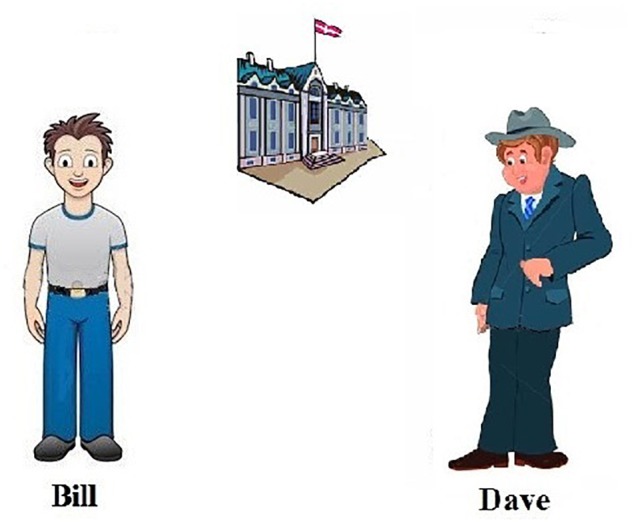
Sample of picture materials.

The visual stimuli were colored cartoon images of four male characters; the names of the characters appeared at the bottom of the image, as illustrated in Figure 1. The position of the characters was counterbalanced across trials – in half of the cases the target picture appeared on the right side of the screen, and in the other half it appeared on the left side. A distractor picture depicting a location was included in the background.

#### Procedure and Coding

In the eye-tracking study, pictures were presented on a monitor using a desktop mounted Eyelink 1000 that records eye movements at a 1000 Hz sampling rate. The eye-tracker was calibrated and validated for each participant by a research assistant. In each trial, a fixation cross appeared in the middle of the screen prior to the display of the pictures. After the fixation, participants saw three pictures on the screen (Target, Competitor and Distractor, see Figure 1) and listened to an experimental/control sentence while the pictures remained on the screen. Participants answered a comprehension question after listening to each sentence. All participants signed a consent form prior to testing. The eye-tracking task took approximately 15 min and was completed by the participants at the beginning of the session.

To analyze the looking behavior in relation to the verbal and visual stimuli presented, two spatial areas of interest (AOI) were selected, corresponding to the size of the pictures presented on the monitor. Eye movements were time-locked to the onset of the pause inserted between the main clause and the subordinate clause (e.g., … in the evening/PAUSE because he …). The eye-movement data were analyzed starting from 200 ms after the onset of the pause to account for the time it takes to program a saccadic eye-movement (Matin et al., 1993), and ending 1500 ms after the onset of the pause. Trials with combined total looking times to the competitor and target of less than 30% of the trial duration (i.e., the 200–1800 ms following the auxiliary) were discarded, amounting to 3% of the data. We analyzed the eye-tracking data based on the bias of the verb present in the main clause (NP1 bias verb vs. NP2 bias verb). Because the effect of congruency was expected to emerge after the second subordinate clause was heard, we did not analyze the data based on congruency. Instead, we focused on predictive processing based on the IC information that was expected to emerge in the early time-windows immediately following the main clause.

In the study by Cozijn et al. (2011), in which a similar experimental design was used, data were aggregated over large time-windows containing segments of the experimental sentences. In the present study, we adopted a more fine-grained analysis by aggregating the eye-movements data in 100 ms time-windows. As shown in the results section, the aggregation in 100 ms time-window gives detailed time-course information about the emergence of the IC effect. For each 100 ms time-window, we calculated and compared the proportion of looks to the competitor and target pictures, aggregated by condition for each participant (see Cozijn et al., 2011, for similar experimental design and data analysis).

For the analysis of the eye-movement data, it has been suggested that using ANOVA over aggregated data has a number of important limitations (e.g., time is not considered in a statistically rigorous manner, and there is no quantification of individual differences; Mirman et al., 2008). Although there is still debate on how to best analyze visual-world data, Growth Curve analysis is currently viewed as a more adequate technique to analyze time-course data (Mirman, 2014). Therefore, growth curve analysis has been adopted to analyze the visual word paradigm results, which allows the modeling of the dependent variable as a function of Time (e.g., Mirman et al., 2008). To compare the speed of processing of bilinguals and monolinguals in the two verb bias conditions, we used a model in which the looks to the target picture are analyzed. In this model, the fixed effects are Verb Bias (NP1 bias verbs vs. NP2 bias verbs), and Group (Bilinguals vs. Monolinguals).

### Results

**FIGURE 2:**
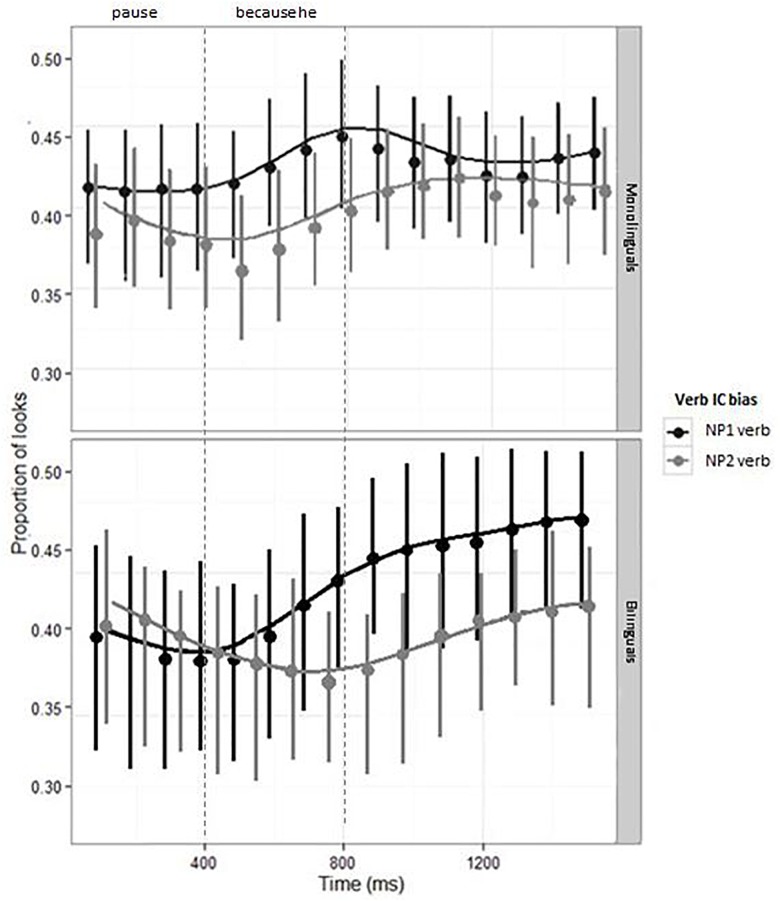
Proportion of looks to the Target, by Verb Bias and Group. Points and range bars show empirical means and 95% confidence intervals. Lines show model estimates. Note: the vertical lines signal the offset of the pause and the onset/offset of the “because he” connector, preceding the disambiguating verb “had/was insulted.”

Figure 2 shows the model estimates and CI for the looks to the target picture in monolingual and bilingual participants. The results are presented by Verb Bias condition (NP1 bias; NP2 bias). Looks to the target picture are time-locked to the onset of the pause inserted before the *because* connector.

Based on visual inspection of the data, Time was coded using a fourth order orthogonal polynomial. The full model is presented in Table 2. The spline components were decorrelated using principal components analysis. For the comparison of the two groups, the dependent variable was the amount of looks to the Target picture. The fixed effects are Verb Bias (NP1 bias verbs vs. NP2 bias verbs), and Group (Bilinguals vs. Monolinguals). We included Random by-Participant intercepts, random slopes and interactions in the By-Subject analysis (see Table 2). In the By-Item analysis, By-Item intercepts, random slopes and interactions were included.

**Table 2:** Fixed effects for models of looks to the target picture in monolinguals and bilinguals.

	β	*SE*	χ*^2^*	*df*	*p*
**By subject analysis**					
Intercept	−4.69556	0.013461			
Time_1	0.002691	0.001083	1.166	4	0.2
Time_2	0.003811	0.003268			
Time_3	0.038196	0.02468			
Time_4					
Verb Bias	−0.00068	0.004681	−0.146	1	0.8
Group	−0.11375	0.027709	1.548	1	0.1
Time_1 × Verb Bias	−0.00127	0.001842	−1.203	4	0.2
Time_2 × Verb Bias	−0.00849	0.007054			
Time_3 × Verb Bias	−0.00881	0.038293			
Time_4 × Verb Bias					
Time_1 × Group	0.001408	0.002241	0.059	4	0.4
Time_2 × Group	0.000402	0.006784			
Time_3 × Group	−0.03881	0.051172			
Time_4 × Group					
Verb Bias × Group	0.006313	0.009665	0.653	1	0.5
Time_1 × Verb Bias × Group	−0.00219	0.003816	−0.574	5	0.5
Time_2 × Verb Bias × Group	−0.03079	0.014628			
Time_3 × Verb Bias × Group	−0.11562	0.079711			
Time_4 × Verb Bias × Group	−0.11562	0.079711			
**By item analysis**					
Intercept	−5.56729	0.02446			
Time_1	−0.09465	0.07891	5.34	4	0.1
Time_2	0.22302	0.20455			
Time_3	1.7736	1.18321			
Time_4	0.15722	0.08456			
Verb Bias	−0.38825	0.23107	1.54	1	0.2
Group	−0.19271	0.01095	1.789	1	0.1
Time_1 × Verb Bias	−0.0045	0.00209	−1.023	4	0.3
Time_2 × Verb Bias	−0.00849	0.00654			
Time_3 × Verb Bias	−0.00790	0.02180			
Time_4 × Verb Bias					
Time_1 × Group	0.00183	0.003456	0.189	4	0.5
Time_2 × Group	0.000234	0.008765			
Time_3 × Group	−0.05988	0.00981			
Time_4 × Group					
Verb Bias × Group	0.04783	0.09321	0.20	1	0.6
Time_1 × Verb Bias × Group	−0.00309	0.008745	−1.034	5	0.3
Time_2 × Verb Bias × Group	−0.09703	0.04618			
Time_3 × Verb Bias × Group	−0.1451	0.06392			
Time_4 × Verb Bias × Group	−0.22784	0.09807			

The analyses did not revealed any main effect or interactions (see Table 2), showing that the amount of looks to the target picture in the two verb bias conditions is very similar in monolinguals and bilinguals^1^. The similarity between the looks to the target in the two groups is also clearly observable in Figure 2. To further clarify the use of the implicit causality bias in bilinguals, in Experiment 2 we conducted a sentence completion task to obtain a measure of the off-line use of the implicit causality bias in sentence continuations.

## Experiment 2: Sentence-Continuation Task

### Participants

The participants were the same as in Experiment 1. One monolingual participant was discarded because she did not understand the task.

### Materials

The sentence-continuation task included sentence fragments that contained a NP1 or NP2 bias verb. The 24 implicit causality verbs used in the eye-tracking experiment (12 NP1 and 12 NP2) were used in the sentence completion task. The sentences included male names (Mike, Brian, John, Eric), one in subject and one in object position, followed by a masculine pronoun, shown in (14). The names were counterbalanced, so that they appeared in half of the sentences in subject position and in the other half in object position.

(14) Mike despised Brian because he

Participants were instructed to complete the sentence with a continuation that sounded natural to them. Forty-eight filler sentences were included in the task that had similar structure as the experimental items, but that did not contain any implicit causality verb. Half of the fillers had a masculine pronoun like the experimental items (e.g., *Mike went skiing with Brian, but he*), the other half included the plural pronoun they (e.g., *Mike and Brian had an argument; later they*). One list was constructed, and then a second list resulted from putting the trials in the opposite order. The task was presented in a word file.

### Coding

Participants’ sentence continuations were scored as NP1, NP2 or unclear. The first author and a native English speaker blind to the purpose of the study scored the productions as NP1 or NP2 continuations. The scoring was then compared until 100% agreement was reached after discussion. A NP1 continuation was scored as such if the pronoun *he* unambiguously referred to the first mentioned entity in the preceding discourse (e.g., Brian inspired Mike because he *had plenty of accomplishments*), while an NP2 continuation required that the pronoun referred to the second mentioned entity in the preceding discourse (e.g., John hated Eric because he *beat him playing video games*). Responses were coded as “unclear” if the referent for the pronoun remained unclear (e.g., John amused Eric at the office because he *knew everyone there*). Thirteen percent of the total responses for monolinguals (12% for NP1 verbs, 13% for NP2 verbs) and 15% of total responses for bilinguals (17% for NP1 and 14% for NP2 verbs) were labeled as unclear, and were discarded from further analysis. Additionally, one trial was discarded from the analysis (verb *punish*) because it was mistakenly repeated twice in the two lists.

**Table 3:** Full model statistics for Experiment 2.

Fixed effects				
	Estimate	St. Error	*t*-value	*p*-value
Intercept	0.63	0.54	1.158	0.24
Verb type	0.38	0.04	−7.926	0.0001
Group	0.28	0.45	0.616	0.53
Verb type ∗ Group	0.57	0.59	0.961	0.33

In the statistical analysis, we analyzed the number of NP1 completions produced by each group in the two verb conditions (NP1 biased vs. NP2 biased) out of the number of completions produced. We used mixed-effects logistic regression (Jaeger, 2008) with Verb Bias condition (2-levels) as fixed effect. In the model, we simplified the random effects structure until convergence was reached (Barr et al., 2013), including random intercepts for participant and item, and participant and item random slope for Verb Bias. We used a stepwise backward inclusion procedure and tested both first-level effects and the interactions between the fixed-effect factors. The number of NP1 completions per each subject and item was coded as 1 or 0 and analyzed using glmer (*lme4* library, Bates and Sarkar, 2007).

### Results

**FIGURE 3:**
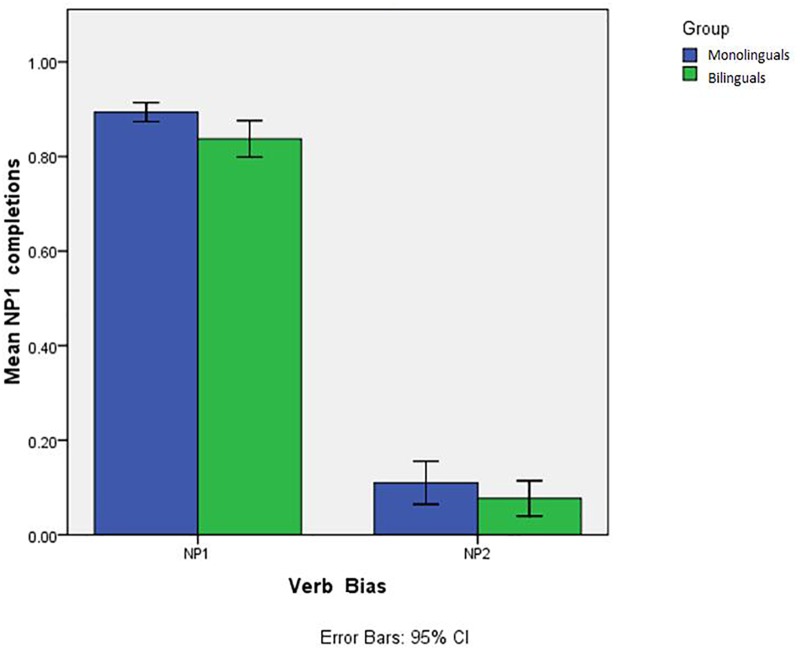
Proportion of NP1 completions in the two verb bias conditions and 95% CI error bars.

Figure 3 illustrates the proportion of NP1 continuations in the two implicit causality verb conditions in monolingual and bilingual participants.

As illustrated in Table 3, the analysis revealed a main effect of Verb Type (ß = 0.38, *SE* = 0.04, *t* = −7.926, *p* < 0.0001), showing more NP1 answers for NP1 verbs compared to NP2 verbs. No other effect or interaction emerged from the analysis.

## Discussion and Conclusion

In Experiment 1, no difference was found between the amounts of looks to the target picture over time between monolinguals and bilinguals. The eye-tracking data suggest that bilinguals do not show general slower referential processing than monolinguals, and their processing of the IC information is comparable. The results of Experiment 2 showed that both monolinguals and bilinguals alike used implicit causality information during a sentence completion task, confirming that bilinguals can use the implicit causality information associated with the verb.

Previous research has hypothesized that weaker predictive abilities in the L2 can be the result of processing difficulties (e.g., Grüter et al., 2016). L2 learners may have fewer processing resources to deploy for prediction, as lexical access and integration of lexical items into the context can be slower and more effortful in the L2 compared to the L1 (Kroll et al., 2012). However, the bilinguals recruited here were very proficient in their L2 (English); recall that they had early exposure to the L2 and had lived in a context of L2 immersion. As observed in the analysis of the eye-tracking results that compared monolinguals and bilinguals, both groups showed comparable looks to the target picture, and no speed of processing differences were observed. Thus, the results demonstrate that highly proficient bilinguals achieve similar expectations for IC verbs in the L2 (English) as monolingual speakers.

When tested on their on-line and off-line expectations, bilinguals demonstrated that their predictions based on implicit causality verbs are equally as strong as in monolingual English speakers.

To conclude, we have shown that for the highly proficient and early exposed bilinguals tested in the present study, no processing cost of activating the verb implicit causality information was observed, suggesting that bilinguals can develop similar online and offline prediction as monolingual speakers.

## Ethics Statement

This study was carried out in accordance with the recommendations of Institutional Review Board of the University of Texas at El Paso with written informed consent from all subjects. All subjects gave written informed consent in accordance with the Declaration of Helsinki. The protocol was approved by the Institutional Review Board of the University of Texas at El Paso.

## Author Contributions

CC was directly responsible for the “Introduction,” “Methods,” “Results,” “Discussion,” and “Conclusion” sections of the manuscript. PD funded and supervised the research reported in the manuscript.

## Conflict of Interest Statement

The authors declare that the research was conducted in the absence of any commercial or financial relationships that could be construed as a potential conflict of interest.

## Footnotes

1 Notice that a similar lack of effects was found when comparing the two groups on the amount of looks to the competitor picture, or by using as dependent measure the difference of looks to target minus competitor.
